# Validation of the Chinese version of trait mental toughness inventory for sport in young basketball players: a confirmatory factor analysis

**DOI:** 10.3389/fpsyg.2026.1772600

**Published:** 2026-05-21

**Authors:** Shaoshen Wang, Ying Shuai, Garry Kuan, Yee Cheng Kueh

**Affiliations:** 1 School of Sports Management, Shandong Sport University, Jinan, Shandong, China; 2 Biostatistics & Research Methodology Unit, School of Medical Sciences, Universiti Sains Malaysia, Kubang Kerian, Kelantan, Malaysia; 3 Exercise and Sports Science Programme, School of Health Sciences, Universiti Sains Malaysia, Kubang Kerian, Kelantan, Malaysia

**Keywords:** basketball, Chinese athletes, confirmatory factor analysis, mental toughness, psychometric properties

## Abstract

**Background:**

Mental toughness represents a fundamental psychological construct crucial for athletic performance, yet its measurement across diverse cultural contexts remains challenging due to the predominance of Western-developed instruments. This study conducted the first comprehensive validation of the Chinese version of the Trait Mental Toughness Inventory for Sport (TMTIS-C) among young basketball players in China.

**Methods:**

A cross-sectional design was employed with 604 secondary school basketball players (ages 12–19) from Shandong Province, China. The TMTIS was culturally adapted through rigorous translation procedures, including forward-backward translation, expert review, and cognitive debriefing. Confirmatory factor analysis was conducted using Mplus 8.7 with Maximum Likelihood Robust estimation, while reliability was assessed through internal consistency and test–retest procedures with a 55-participant subsample.

**Results:**

The three-factor structure (Positive Effort, Antipressure, and Endurance) demonstrated excellent model fit in both initial (CFI = 0.959, TLI = 0.956, RMSEA = 0.04) and final models (CFI = 0.965, TLI = 0.962, RMSEA = 0.037). All factor loadings exceeded 0.60, with composite reliability values ranging from 0.871 to 0.950. Convergent and discriminant validity were confirmed through average variance extracted (0.542–0.600) and Fornell–Larcker criterion. Test–retest reliability coefficients were excellent across all dimensions (ICC = 0.945–0.973).

**Conclusion:**

The TMTIS-C represents a psychometrically robust and culturally appropriate instrument for assessing mental toughness in Chinese young athletes, providing researchers and practitioners with a reliable tool for cross-cultural mental toughness research and supporting the development of culturally sensitive interventions in Asian sporting contexts.

## Introduction

Mental toughness represents a critical psychological attribute that enables athletes to maintain optimal performance under pressure, adversity, and challenging circumstances (Jones et al., 2007). Originally conceptualized by Cattell et al. (1955) as a personality trait influenced by environmental and cultural factors, mental toughness has evolved into a multifaceted construct that encompasses cognitive, emotional, and behavioral components essential for athletic success. The significance of mental toughness in competitive sports has been consistently demonstrated across various athletic populations, with research indicating that approximately 90% of studies support the notion that athletes with higher levels of mental toughness perform better than their counterparts (Cowden, 2017). Recent research has continued to demonstrate the effectiveness of mental toughness interventions, with systematic reviews showing significant improvements in athletes’ psychological resilience and competitive performance (Arthur et al., 2015).

The conceptual development of mental toughness has progressed through several distinct phases. Early pioneering work by Loehr (1982) brought mental toughness to the forefront of sports psychology, proposing that elite athletes maintain composure through specific cognitive strategies encompassing self-confidence, negative energy control, and attention regulation. Subsequent research by Clough et al. (2002) established a foundational framework identifying four core components: commitment, control, challenge, and self-confidence. This conceptualization was further refined by Jones et al. (2007), who conducted comprehensive interviews with elite athletes, coaches, and psychologists to identify 30 attributes across four dimensions: attitude/mindset, training, competition, and post-competition processing. Contemporary understanding has expanded to include the role of mindfulness-based approaches, with emerging evidence suggesting that acceptance-based interventions enhance mental toughness through improved present-moment awareness and reduced experiential avoidance (Gardner and Moore, 2007).

Contemporary research has demonstrated that mental toughness significantly impacts athletic performance across diverse sporting contexts. Meta-analytical evidence reveals moderate to high correlations between mental toughness and sports performance, with these relationships being influenced by factors such as age, sports category, and measurement approaches (Hsieh et al., 2023). Furthermore, intervention studies have shown that mental toughness training produces large effect sizes, with psychological interventions being particularly effective in enhancing this construct (Stamatis et al., 2020). Recent longitudinal research has also demonstrated that combined psychological skills training and mindfulness-based interventions significantly improve mental toughness, competitive anxiety management, and coping skills in athletes (Vella-Fondacaro and Romano-Smith, 2023). Research has also highlighted the importance of developmental considerations, with studies showing that mental toughness training programs can be effectively implemented with youth athletes to improve their psychological skills and competitive performance (Connaughton et al., 2008).

Despite the recognized importance of mental toughness in athletic performance, significant challenges remain in its accurate measurement and assessment. The subjective nature of this psychological construct has necessitated the development of various psychometric instruments, each with distinct theoretical foundations and measurement approaches. Early attempts at measurement, such as the Psychological Performance Inventory (PPI) by Loehr (1984), faced criticism for structural validity issues. Subsequent developments included the multidimensional MTQ-48 scale by Clough et al. (2002), which has been refined into shorter versions (MTQ-18 and MTQ-10) to address psychometric concerns and improve practical applicability. Contemporary psychometric research has emphasized the importance of rigorous validation procedures, with studies highlighting that many psychological instruments in sport require careful factorial validation to ensure their structural integrity (Marsh et al., 2014).

The evolution of mental toughness measurement has increasingly emphasized the need for sport-specific and culturally sensitive instruments. Recent advancements include specialized questionnaires such as the Australian Football Mental Toughness Inventory (AfMTI) and Cricket Mental Toughness Inventory (CMTI), which consider the unique aspects of particular sports contexts. The Mental Toughness Scale (MTS) by Madrigal et al. (2013) and the Mental Toughness Index (MTI) by Gucciardi et al. (2015) have shown promise in terms of validity and reliability, though the selection of appropriate instruments remains dependent on specific sporting contexts and target populations. Cross-cultural research has particularly emphasized the importance of cultural adaptation, with studies demonstrating significant cultural differences in the expression and measurement of psychological constructs, necessitating careful validation across different populations (Byrne, 2008).

Among the various available instruments, the Trait Mental Toughness Inventory for Sport (TMTIS) developed by Huang (2003) presents a particularly relevant tool for assessing mental toughness in Asian athletic populations. The behavioral focus of the TMTIS aligns with contemporary sport psychology approaches that emphasize observable performance-related behaviors rather than purely cognitive or personality-based attributes (Weinberg and Gould, 2023).

The cultural specificity of mental toughness measurement presents important considerations for cross-cultural research. While Western-developed scales have dominated the literature, there is growing recognition of the need for culturally appropriate instruments that account for diverse cultural contexts and sporting traditions. The TMTIS, developed within an Asian cultural context, offers potential advantages for assessing mental toughness in Chinese athletic populations, potentially providing better cultural sensitivity compared to Western-developed scales. Contemporary guidelines for cross-cultural adaptation emphasize that successful instrument adaptation requires comprehensive procedures including translation, back-translation, and cultural adaptation to ensure conceptual and semantic equivalence (Wild et al., 2005).

Basketball, as a dynamic team sport characterized by frequent high-pressure situations, rapid decision-making demands, and intense competitive environments, presents unique challenges that require specific mental toughness attributes. Young basketball players, in particular, face developmental challenges that may influence their mental toughness profiles and require age-appropriate assessment approaches. The three dimensions of the TMTIS appear particularly relevant to basketball performance: active endeavour relates to the persistent effort required for skill development and game execution, stress resistance addresses the ability to maintain performance under competitive pressure, and endurance encompasses the sustained mental focus required throughout extended training and competition periods. Research in basketball has demonstrated that psychological factors, including mental toughness, play crucial roles in performance outcomes, with studies showing significant relationships between mental skills and basketball performance measures (Katartzi and Vlachopoulos, 2011).

The importance of mental toughness in youth sport development has been increasingly recognized, with research demonstrating that psychological skills training can significantly improve young athletes’ competitive performance and psychological wellbeing (Visek et al., 2009). Furthermore, studies indicate that the development of mental toughness in youth athletes requires consideration of age-specific factors and developmental-appropriate interventions.

Despite its potential utility, the TMTIS has received limited attention in the international literature, and its psychometric properties require thorough validation in diverse populations. Confirmatory factor analysis (CFA) represents the gold standard for validating the factorial structure of psychological instruments, providing rigorous statistical examination of theoretical models and their empirical support. The application of CFA to the TMTIS would contribute significantly to the mental toughness literature by providing empirical evidence for its theoretical structure and practical utility in athletic assessment. Contemporary standards for structural equation modeling in sport psychology research emphasize the importance of appropriate model specification, adequate sample sizes, and the use of multiple fit indices to evaluate model adequacy (Kline, 2023).

### Study purpose and objectives

The primary purpose of this study was to conduct a comprehensive psychometric evaluation of the Trait Mental Toughness Inventory for Sport (TMTIS-C) among young basketball players in Shandong Province, China. Specifically, this research aimed to: (1) examine the factorial validity of the TMTIS-C three-factor structure (active endeavour, stress resistance, and endurance) through confirmatory factor analysis; (2) assess the internal consistency and reliability of the TMTIS-C subscales; (3) evaluate the construct validity of the instrument through convergent and discriminant validity analyses; and (4) provide empirical evidence for the psychometric soundness and cultural appropriateness of the TMTIS-C as a measure of mental toughness in Chinese young athletes.

The findings of this study will contribute to the broader understanding of mental toughness measurement in sports psychology, particularly within Asian cultural contexts. By validating the TMTIS through rigorous statistical analysis, this research will provide practitioners and researchers with a reliable and culturally sensitive instrument for assessing mental toughness in young athletes, ultimately supporting the development of more effective mental skills training programs and performance enhancement interventions.

## Materials and methods

### Participants

This investigation utilized a cross-sectional research framework to evaluate the psychometric characteristics of the Chinese adaptation of the Trait Mental Toughness Inventory for Sport (TMTIS-C). This study employed a three-stage stratified random sampling procedure to recruit participants (Etikan and Bala, 2017). In Stage 1, the target population—all adolescent basketball players in middle and high schools across Shandong Province—was divided into mutually exclusive strata based on geographic location, with each city treated as a single stratum. In Stage 2, simple random sampling was used within each stratum to select two schools: one middle school and one high school, ensuring representation across both geographic regions and educational levels. In Stage 3, a complete roster of eligible basketball team members was obtained from each selected school, from which 20 participants were randomly selected. This multi-stage process ensured that players from diverse cities and school types were systematically represented in the final sample. The recruitment approach guaranteed representation across varying competitive standards and athletic development backgrounds. A total of 605 questionnaire sets were distributed to eligible participants. Of these, 604 were returned fully completed, yielding a response rate of 99.8%. All returned questionnaires were deemed usable for analysis.

Inclusion parameters for study participation encompassed: (1) active registration in secondary school basketball programs; (2) consistent engagement in structured basketball training and competitive activities; (3) demonstrated competency in Mandarin Chinese reading comprehension; and (4) willingness to participate with appropriate informed consent documentation. Exclusion criteria applied to students reporting significant health impairments that compromised regular basketball participation during the preceding six-month period or those lacking competitive exposure at municipal competition levels or above.

### Sample size calculation

Statistical power requirements for confirmatory factor analysis were determined utilizing the web-based computational tool developed by Arifin (2022), incorporating guidelines from contemporary structural equation modeling literature. The calculation integrated the following analytical specifications: projected CFI value of 0.95, three-dimensional construct containing 16, 11, and 5 indicators respectively, anticipated factor loadings of 0.5, inter-dimensional correlations of 0.2, statistical significance threshold (*α*) of 0.05 (bidirectional), and target statistical power (1 − *β*) of 90%. After adjusting for anticipated participant dropout (15%), the minimal necessary sample size was established at 586 individuals. The final recruited sample (*N* = 604) considerably surpassed this requirement, ensuring adequate parameter estimation precision and reliable factor extraction outcomes.

### Measures

#### Trait Mental Toughness Inventory for Sport (TMTIS)

The TMTIS is a validated multidimensional instrument originally developed by Huang (2003) in Taiwan to assess stable mental toughness characteristics in athletic populations. The scale development employed a systematic multi-phase approach, progressing from initial qualitative research through exploratory factor analysis (EFA) on 289 university athletes to confirmatory factor analysis (CFA) validation with 413 participants.

The final 32-item version measures three core dimensions of mental toughness: (1) Positive Effort (16 items)—reflecting proactive engagement and perseverance in training and competition; (2) Antipressure (11 items)—capturing ability to manage high-pressure situations effectively; and (3) Endurance (5 items)—representing capacity to withstand physical discomfort and training demands. Items are rated on a 5-point Likert scale (1 = strongly disagree to 5 = strongly agree).

The TMTIS demonstrates robust psychometric properties. Internal consistency reliability is excellent across all subscales (Cronbach’s *α* = 0.93 for Positive Effort, 0.90 for Antipressure, 0.84 for Endurance). Construct validity was established through CFA, revealing acceptable model fit indices (*χ*^2^/df = 2.24, CFI = 0.91, GFI = 0.86, RMSEA = 0.06). Inter-factor correlations ranged from 0.37 to 0.63, supporting the distinctiveness of the three dimensions while confirming their theoretical relatedness (Huang, 2003).

### Ethics and procedures

This investigation obtained ethical clearance from the Universiti Sains Malaysia Human Research Ethics Committee (USM/JEPeM/22050298). The cultural adaptation of the TMTIS into Simplified Chinese (TMTIS-C) employed a comprehensive translation and validation protocol conforming to established cross-cultural adaptation frameworks.

The linguistic adaptation commenced with dual independent forward translations conducted by bilingual scholars with expertise in both English and Mandarin Chinese. Subsequently, these translators collaborated to develop a preliminary Chinese version through systematic comparison and consensus-building discussions. An expert panel consisting of three sports psychologists, two sports science researchers, and two physical education specialists assessed this initial draft for content validity and cultural appropriateness. Additionally, two Traditional Chinese experts were consulted to ensure accurate adaptation from the original Taiwanese version. Expert recommendations guided essential modifications in terminology and linguistic expression.

Following expert evaluation, two distinct bilingual researchers performed back-translation of the modified Chinese version into English. The back-translated material was systematically compared against the original TMTIS to verify conceptual and semantic consistency. Any identified discrepancies underwent iterative discussion and revision until achieving acceptable alignment with the source instrument. To strengthen face validity, the refined translation was subjected to cognitive debriefing with 12 native Chinese speakers, who assessed clarity, comprehensibility, and cultural relevance. A preliminary investigation involving 15 adolescent basketball players subsequently validated the instrument’s readability and practical applicability.

Data acquisition spanned from February 2023 to July 2023. The research team distributed informed consent documentation to prospective participants and their parents or legal guardians, detailing the study’s objectives, voluntary participation principles, and confidentiality protocols. Following written consent acquisition, participants accessed the TMTIS-C questionnaire via the Sojump platform using personal or parental electronic devices. Before questionnaire administration, researchers delivered standardized instructions emphasizing authentic responses and clarifying the absence of correct or incorrect answers. The complete data collection protocol maintained compliance with ethical standards governing adolescent research participation.

Test–retest reliability assessment utilized a randomly selected subsample of 55 participants from the primary cohort, who completed the TMTIS-C on two occasions separated by a 2-week interval. This subsample size was calculated using Arifin (2022) computational tool, incorporating anticipated ICC of 0.80, minimum acceptable ICC of 0.60, with 80% statistical power and 10% attrition adjustment.

### Statistical analysis

Preliminary data examination assessed univariate and multivariate distributional characteristics utilizing SPSS 28.0 (IBM Corp, Armonk, NY, USA). Univariate normality was evaluated through Kolmogorov–Smirnov and Shapiro–Wilk procedures (Ghasemi and Zahediasl, 2012), whereas multivariate normality was determined via Mardia’s tests examining skewness and kurtosis parameters. These statistical evaluations were supplemented by visual examination of histogram distributions and Chi-square versus Mahalanobis distance scatterplots, consistent with guidelines outlined by Tabachnick et al. (2019).

Confirmatory Factor Analysis (CFA) was executed using Mplus 8.7 employing Maximum Likelihood Robust (MLR) estimation, chosen to accommodate non-normal data distributions as suggested by Muthén and Muthén (2017). Model adequacy was assessed through multiple goodness-of-fit indices adhering to thresholds established by Hu and Bentler (1999): Comparative Fit Index (CFI > 0.95), Tucker–Lewis Index (TLI > 0.95), Root Mean Square Error of Approximation (RMSEA < 0.06, including 90% confidence intervals), and Standardized Root Mean Square Residual (SRMR < 0.08). Factor loadings surpassing 0.40 were deemed satisfactory according to Hair et al. (2012).

Model refinements were executed through a systematic stepwise methodology. Residual correlations were incorporated sequentially based on modification indices, with the refinement process terminated upon achieving satisfactory fit thresholds (CFI ≥ 0.95, TLI ≥ 0.95) to preserve model parsimony and prevent overfitting.

Modification indices (MI) were scrutinized to inform model enhancements, with values exceeding 10.0 considered for potential structural adjustments, following Byrne (2013). Only theoretically defensible modifications were incorporated, emphasizing residual correlations between items within identical factors or conceptually associated items across factors. The highest MI values were prioritized in the sequential modification procedure, with each adjustment evaluated for both statistical enhancement and theoretical validity prior to implementation.

Construct validity evaluation encompassed convergent and discriminant validity assessments. Convergent validity was demonstrated through factor loadings (>0.50), Average Variance Extracted (AVE > 0.50), and Composite Reliability (CR > 0.70) following Hair et al. (2012). Discriminant validity was examined using the Fornell–Larcker criterion (Fornell and Larcker, 1981), requiring the square root of AVE for each construct to surpass its correlations with remaining constructs.

Reliability assessment incorporated internal consistency and temporal stability evaluations. Internal consistency was determined through CR, with values exceeding 0.70 indicating adequate reliability (Taber, 2018). Test–retest reliability was examined using two-way mixed effects Intraclass Correlation Coefficients (ICC) following Koo and Li (2016), with a 2-week interval between initial and subsequent administrations, with interpretations as: poor (<0.50), moderate (0.50–0.75), good (0.75–0.90), and excellent (>0.90).

The two-way mixed effects ICC model was selected because the two assessment occasions were fixed conditions applied uniformly to all participants, while participants constituted a random sample from the target population (Shrout and Fleiss, 1979). Although TMTIS-C items employ a Likert-type response format, ICC analyses were conducted on composite subscale scores rather than individual item responses. Composite scores derived from multiple ordinal items approximate interval-level measurement, thereby satisfying the distributional assumptions underlying ICC computation (Koo and Li, 2016; Weir, 2005).

## Results

### Descriptive statistics

The study comprised 604 secondary school students. Demographic characteristics of the full sample are presented in Table 1. In summary, the sample was approximately evenly split by gender (males: 49.8%; females: 50.2%), with a mean age of 15.53 years (SD = 1.42) and predominant representation from senior secondary school levels (senior 1–3: 83.6% combined). Approximately 71.7% of participants held a recognised sport classification at junior grade or above (Table 1).

**Table 1:** Demographic information and frequency of participants.

Category	Frequencies	Percentage	Mean (SD)
Gender			
Male	301	49.8	
Female	303	50.2	
Grade			
Junior 1	34	5.6	
Junior 2	26	4.3	
Junior 3	39	6.5	
Senior 1	169	28	
Senior 2	157	26	
Senior 3	179	29.6	
Age			15.53 (1.42)
Training years			
Less than 1 year	72	11.9	
1 year	209	34.6	
2 years	188	31.1	
3 years	88	14.6	
More than 3 years	47	7.8	
Sport level			
None	171	28.3	
Junior grade	184	30.5	
Level 3	140	23.2	
Level 2	67	11.1	
Level 1	30	5	
Master	12	2	

Table 2 shows the distribution of the items’ scores for the Chinese version of the TMTIS. The mean (SD) scores for all items ranged between 3.14 (1.3) for TMTIS11 and 3.76 (1.43) for TMTIS32. Specifically, most participants answered “1 (never)” for TMTIS18 (22.7%), “2 (rarely)” for TMTIS32 (22.0%), “3 (sometimes)” for TMTIS11 (24.8%), “4 (often)” for TMTIS13 (48.7%), and “5 (always)” for TMTIS32 (47.2%). The overall total and average score mean (SD) of TMTIS were calculated as part of this study, reflecting the participants’ levels of trait mental toughness. The total mean scores (SD) for Positive effort, Antipressure, and Endurance were 54.56 (16.73), 37.74 (11.62), and 16.46 (5.22), respectively. The average scores (SD) for Positive effort, Antipressure, and Endurance were 3.41 (1.05), 3.43 (1.06), and 3.29 (1.04), respectively.

**Table 2:** Distribution of the items’ score for Chinese version of TMTIS scale.

Items	Mean (SD)	(1) *n* (%)	(2) *n* (%)	(3) *n* (%)	(4) *n* (%)	(5) *n* (%)
TMTIS1	3.38 (1.40)	93 (15.4)	88 (14.6)	72 (11.9)	198 (32.8)	153 (25.3)
TMTIS2	3.29 (1.46)	132 (21.9)	50 (8.3)	63 (10.4)	227 (37.6)	132 (21.9)
TMTIS3	3.69 (1.40)	51 (8.4)	130 (21.5)	13 (2.2)	169 (28.0)	241 (39.9)
TMTIS4	3.41 (1.43)	84 (13.9)	101 (16.7)	100 (16.6)	124 (20.5)	195 (32.3)
TMTIS5	3.43 (1.35)	70 (11.6)	95 (15.7)	116 (19.2)	149 (24.7)	174 (28.8)
TMTIS6	3.40 (1.38)	83 (13.7)	88 (14.6)	108 (17.9)	157 (26.0)	168 (27.8)
TMTIS7	3.20 (1.29)	89 (14.7)	92 (15.2)	130 (21.5)	195 (32.3)	98 (16.2)
TMTIS8	3.27 (1.42)	125 (20.7)	57 (9.4)	62 (10.3)	247 (40.9)	113 (18.7)
TMTIS9	3.40 (1.40)	81 (13.4)	98 (16.2)	104 (17.2)	142 (23.5)	179 (29.6)
TMTIS10	3.36 (1.38)	82 (13.6)	95 (15.7)	116 (19.2)	147 (24.3)	164 (27.2)
TMTIS11	3.14 (1.30)	102 (16.9)	77 (12.7)	150 (24.8)	187 (31.0)	88 (14.6)
TMTIS12	3.67 (1.41)	58 (9.6)	123 (20.4)	15 (2.5)	170 (28.1)	238 (39.4)
TMTIS13	3.34 (1.45)	134 (22.2)	47 (7.8)	15 (2.5)	294 (48.7)	114 (18.9)
TMTIS14	3.67 (1.43)	67 (11.1)	114 (18.9)	8 (1.3)	180 (29.8)	235 (38.9)
TMTIS15	3.38 (1.38)	77 (12.7)	98 (16.2)	118 (19.5)	140 (23.2)	171 (28.3)
TMTIS16	3.36 (1.40)	85 (14.1)	94 (15.6)	113 (18.7)	140 (23.2)	172 (28.5)
TMTIS17	3.15 (1.33)	109 (18.0)	72 (11.9)	143 (23.7)	182 (30.1)	98 (16.2)
TMTIS18	3.26 (1.45)	137 (22.7)	44 (7.3)	68 (11.3)	237 (39.2)	118 (19.5)
TMTIS19	3.68 (1.42)	60 (9.9)	121 (20)	11 (1.8)	172 (28.5)	240 (39.7)
TMTIS20	3.41 (1.42)	92 (15.2)	78 (12.9)	106 (17.5)	146 (24.2)	182 (30.1)
TMTIS21	3.39 (1.40)	83 (13.7)	94 (15.6)	105 (17.4)	147 (24.3)	175 (29.0)
TMTIS22	3.27 (1.31)	77 (12.7)	105 (17.4)	118 (19.5)	185 (30.6)	119 (19.7)
TMTIS23	3.28 (1.44)	129 (21.4)	52 (8.6)	63 (10.4)	239 (39.6)	121 (20.0)
TMTIS24	3.58 (1.43)	62 (10.3)	119 (19.7)	68 (11.3)	114 (18.9)	241 (39.9)
TMTIS25	3.32 (1.32)	72 (11.9)	111 (18.4)	102 (16.9)	187 (31.0)	132 (21.9)
TMTIS26	3.40 (1.26)	55 (9.1)	126 (20.9)	63 (10.4)	245 (40.6)	115 (19.0)
TMTIS27	3.45 (1.58)	134 (22.2)	47 (7.8)	67 (11.1)	128 (21.2)	228 (37.7)
TMTIS28	3.36 (1.40)	88 (14.6)	90 (14.9)	110 (18.2)	149 (24.7)	167 (27.6)
TMTIS29	3.48 (1.28)	59 (9.8)	122 (20.2)	11 (1.8)	293 (48.5)	119 (19.7)
TMTIS30	3.46 (1.57)	130 (21.5)	51 (8.4)	63 (10.4)	129 (21.4)	231 (38.2)
TMTIS31	3.42 (1.35)	69 (11.4)	102 (16.9)	111 (18.4)	151 (25.0)	171 (28.3)
TMTIS32	3.76 (1.43)	48 (7.9)	133 (22.0)	22 (3.6)	116 (19.2)	285 (47.2)

1 = never, 2 = rarely, 3 = sometimes, 4 = often, 5 = always; SD = standard deviation.

### Confirmatory factor analysis

Confirmatory factor analysis was conducted to evaluate the factorial structure of the TMTIS-C using a three-factor model comprising Positive Effort (16 items), Antipressure (11 items), and Endurance (5 items). Two models were examined: an initial model without correlated residuals and a final model incorporating theoretically justified residual correlations based on modification indices.

The initial three-factor model demonstrated acceptable fit to the data, with fit indices approaching recommended thresholds (CFI = 0.959, TLI = 0.956, SRMR = 0.034, RMSEA = 0.04 [90% CI: 0.036, 0.044]). Examination of modification indices revealed three significant correlations exceeding the threshold of 10.0: TMTIS32 WITH TMTIS1 (M.I. = 32.609), TMTIS32 WITH TMTIS19 (M.I. = 23.123), and TMTIS19 WITH TMTIS1 (M.I. = 16.223). These correlations were theoretically justifiable as all items belonged to the Positive Effort dimension and shared similar content domains.

The final model, incorporating these three residual correlations, yielded improved fit indices (CFI = 0.965, TLI = 0.962, SRMR = 0.031, RMSEA = 0.037 [90% CI: 0.033, 0.041]), all exceeding recommended criteria for good model fit. As presented in Table 3, the final model demonstrated superior fit across all indices compared to the initial model.

**Table 3:** CFA fit indices for the TMTIS-C (initial and final models).

Model	CFI	TLI	SRMR	RMSEA (90%CI)
Initial model^a^	0.959	0.956	0.032	0.040 (0.036, 0.044)
Final model^b^	0.965	0.962	0.031	0.037 (0.033, 0.041)

Model^a^ with correlated items’ residual: TMTIS32 WITH TMTIS1; TMTIS32 WITH TMTIS19; TMTIS19 WITH TMTIS1.Model^b^ with correlated items’ residual: TMTIS32 WITH TMTIS1; WITH TMTIS19; TMTIS19 WITH TMTIS1.

**Figure 1:**
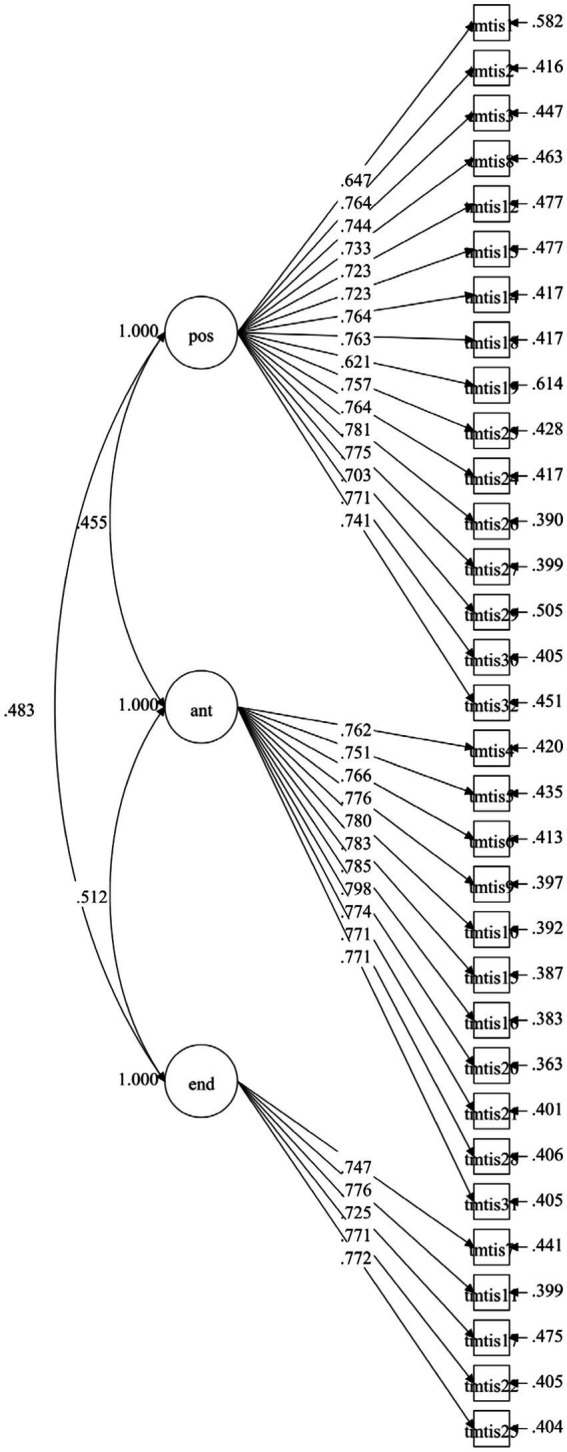
CFA diagram of the TMTIS-C initial model. pos, Positive effort; ant, Antipressure; end, Endurance.

**Figure 2:**
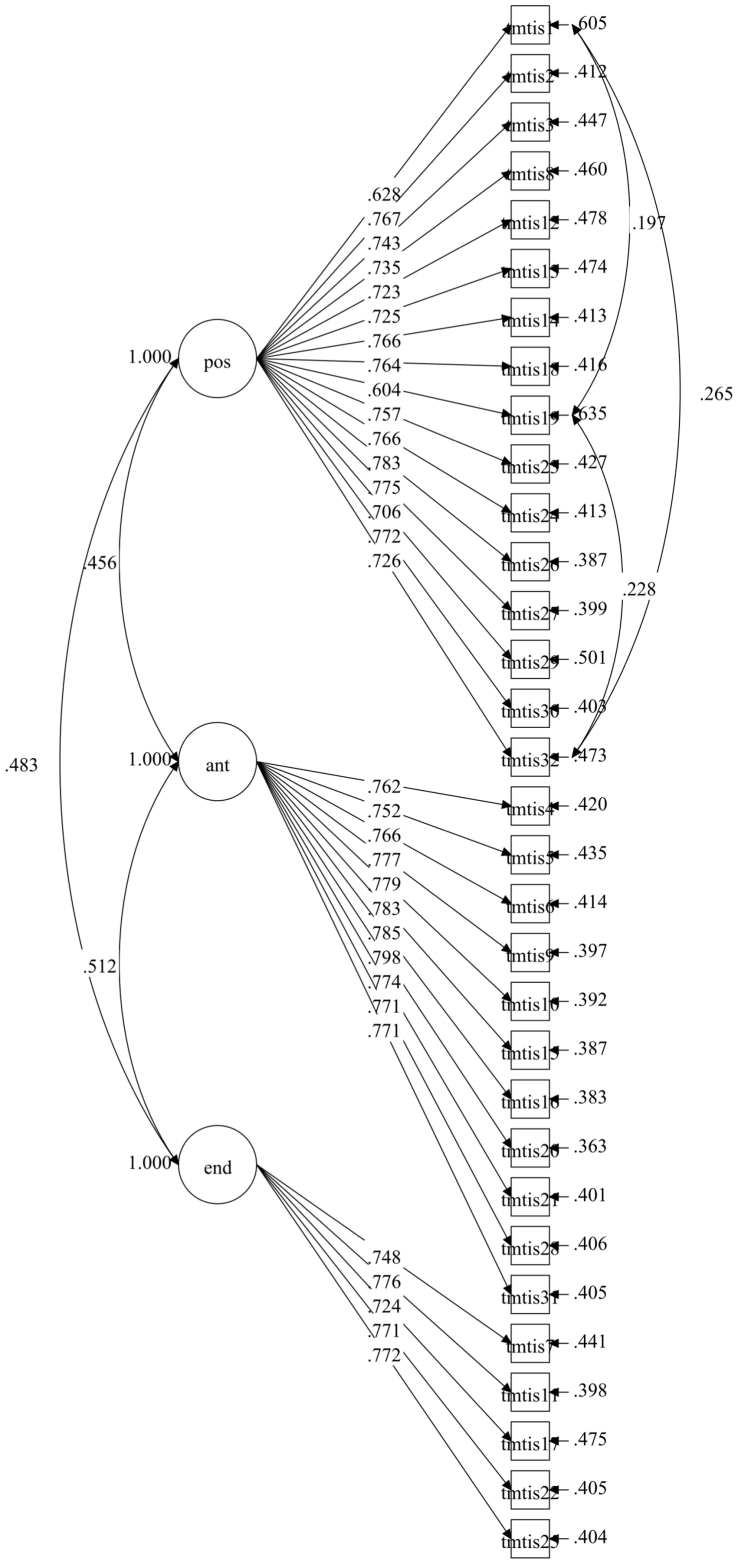
CFA diagram of the TMTIS-C final model. pos, Positive effort; ant, Antipressure; end, Endurance.

Table 3 presents the CFA fit indices for both models, while Figure 1 CFA Diagram of the TMTIS-C Initial model and Figure 2 CFA Diagram of the TMTIS-C Final model illustrate the structural diagrams for the initial and final models, respectively. As shown in Table 4, standardized factor loadings for both models remained robust and consistent. In the final model, factor loadings for the Positive Effort dimension ranged from 0.604 to 0.783, with all loadings exceeding the minimum threshold of 0.40. The Antipressure dimension demonstrated strong factor loadings ranging from 0.752 to 0.798, while the Endurance dimension exhibited loadings between 0.724 and 0.776. All factor loadings were statistically significant (*p* < 0.001) and exceeded the recommended criterion of 0.50 for adequate convergent validity.

**Table 4:** Factor loadings of the TMTIS-C for initial and final models.

Factors/items	Factor loading	
	Initial model	Final model
Positive effort		
TMTIS1	0.647	0.628
TMTIS2	0.764	0.767
TMTIS3	0.744	0.743
TMTIS8	0.733	0.735
TMTIS12	0.723	0.723
TMTIS13	0.723	0.725
TMTIS14	0.764	0.766
TMTIS18	0.763	0.764
TMTIS19	0.621	0.604
TMTIS23	0.757	0.757
TMTIS24	0.764	0.766
TMTIS26	0.781	0.783
TMTIS27	0.775	0.775
TMTIS29	0.703	0.706
TMTIS30	0.771	0.772
TMTIS32	0.741	0.726
Antipressure		
TMTIS4	0.762	0.762
TMTIS5	0.751	0.752
TMTIS6	0.766	0.766
TMTIS9	0.776	0.777
TMTIS10	0.780	0.779
TMTIS15	0.783	0.783
TMTIS16	0.785	0.785
TMTIS20	0.798	0.798
TMTIS21	0.774	0.774
TMTIS28	0.771	0.771
TMTIS31	0.771	0.771
Endurance		
TMTIS7	0.747	0.748
TMTIS11	0.776	0.776
TMTIS17	0.725	0.724
TMTIS22	0.771	0.771
TMTIS25	0.772	0.772

### Convergent and discriminant validity

Convergent and discriminant validity of the TMTIS-C were assessed through examination of composite reliability (CR), average variance extracted (AVE), and inter-construct correlations. As presented in Table 5 Correlation Matrix and Discriminant Validity of the TMTIS-C (Initial and Final models), both the initial and final models demonstrated strong evidence of convergent validity across all dimensions.

**Table 5:** Correlation matrix and discriminant validity of the TMTIS-C (initial and final models).

Initial	CR	AVE	POS	ANT	END
POS	0.950	0.543	0.737		
ANT	0.943	0.600	0.455*	0.774	
END	0.871	0.575	0.483*	0.512*	0.758

Final	CR	AVE	POS	ANT	END
POS	0.950	0.542	0.736		
ANT	0.943	0.600	0.456*	0.774	
END	0.871	0.575	0.483*	0.512*	0.759

*p < 0.05 CR, composite reliability; AVE, average variance extracted; POS, positive effort; ANT, Antipressure; END, Endurance.

For convergent validity, all three dimensions exceeded the recommended thresholds established by Hair et al. (2012) Composite reliability values were consistently high across both models: Positive Effort (CR = 0.950), Antipressure (CR = 0.943), and Endurance (CR = 0.871), all surpassing the minimum criterion of 0.70. Average variance extracted values also met the recommended threshold of 0.50, with Positive Effort (AVE = 0.543 in initial model, 0.542 in final model), Antipressure (AVE = 0.600), and Endurance (AVE = 0.575) demonstrating adequate convergent validity.

Discriminant validity was evaluated using the Fornell–Larcker criterion, which requires the square root of AVE for each construct to exceed its correlations with other constructs. The diagonal values in Table 5 represent the square roots of AVE: Positive Effort (0.737 in initial model, 0.736 in final model), Antipressure (0.774), and Endurance (0.758 in initial model, 0.759 in final model). All diagonal values exceeded the corresponding inter-construct correlations, confirming discriminant validity.

Inter-construct correlations were moderate and statistically significant (*p* < 0.05), ranging from 0.455 to 0.512 across both models. The correlation between Positive Effort and Antipressure was 0.455 in the initial model and 0.456 in the final model. The correlation between Positive Effort and Endurance remained stable at 0.483 in both models, while the correlation between Antipressure and Endurance was consistent at 0.512. These moderate correlations indicate that while the three dimensions are theoretically related components of mental toughness, they represent distinct constructs with adequate discriminant validity.

The minimal differences in validity indices between the initial and final models (with changes ≤ 0.001) demonstrate the stability of the construct validity across model specifications. The final model maintained the same high levels of convergent and discriminant validity as the initial model, confirming that the incorporation of residual correlations did not compromise the distinctiveness of the three mental toughness dimensions.

Overall, the TMTIS-C demonstrated robust construct validity, with all dimensions meeting established criteria for both convergent and discriminant validity. These findings support the theoretical three-factor structure and provide evidence for the scale’s ability to reliably distinguish between different aspects of mental toughness in young basketball players.

### Test–retest reliability

The test–retest reliability of the TMTIS-C was evaluated using the Intraclass Correlation Coefficient (ICC) values for the three subscales, indicating excellent stability over time. The ICC values were as follows: Positive Effort (0.968), Antipressure (0.973), and Endurance (0.945). These values demonstrate excellent reliability, with all ICC values exceeding 0.90, indicating excellent reliability according to Koo and Li (2016) criteria (Table 6).

**Table 6:** Intraclass correlation coefficients (ICC) for the TMTIS-C.

Dimension	ICC
Positive effort+	0.968
Antipressure	0.973
Endurance	0.945

## Discussion

The present study provides robust empirical evidence for the factorial validity and psychometric soundness of the TMTIS-C among young Chinese basketball players. The successful validation of the three-factor structure (Positive Effort, Antipressure, and Endurance) through confirmatory factor analysis represents a significant contribution to the cross-cultural measurement of mental toughness in sports psychology, particularly within Asian contexts where culturally sensitive instruments remain scarce. This validation addresses important gaps identified in the literature regarding the need for culturally adapted psychological measures in sport psychology research (Sue and Chang, 2003).

The superior fit indices achieved by the final model align with contemporary standards for structural equation modeling, corroborating the theoretical framework originally proposed by Huang (2003). These findings extend previous validation work by demonstrating that the TMTIS maintains its psychometric integrity when adapted from Traditional Chinese to Simplified Chinese contexts. The minimal modifications required (three residual correlations within the Positive Effort dimension) suggest that the instrument’s theoretical structure remains stable across Chinese populations, supporting its cross-regional applicability within similar cultural contexts. The successful application of confirmatory factor analysis in this study follows current best practices in sport psychology research, which emphasize rigorous statistical validation procedures (Thompson, 2004).

The excellent internal consistency and test–retest reliability coefficients observed across all dimensions surpass those reported in many Western-developed mental toughness instruments, including the widely-used MTQ-48 and SMTQ scales (Clough et al., 2002; Sheard et al., 2009). The particularly strong reliability for the Positive Effort dimension (ICC = 0.968) may reflect the cultural emphasis on persistence and effort within Chinese athletic contexts, consistent with research highlighting the importance of cultural differences in self-improvement motivations in East Asian cultures (Heine et al., 2001). This cultural alignment potentially enhances the instrument’s sensitivity to mental toughness variations within Chinese populations compared to Western alternatives. Research in cultural psychology has consistently demonstrated that psychological constructs manifest differently across cultures, with collectivistic cultures often emphasizing effort and persistence over individual achievement orientations (Hofstede, 2001).

This finding is particularly important given criticisms of earlier instruments that failed to adequately differentiate between mental toughness components (Andersen, 2012). The clear discriminant validity supports the multidimensional nature of mental toughness and provides evidence against unidimensional conceptualizations that have dominated some areas of the literature. The robust convergent and discriminant validity evidence addresses longstanding concerns regarding the construct validity of mental toughness measures (Gucciardi and Jones, 2012). The moderate inter-factor correlations (0.455–0.512) demonstrate that while the three dimensions are conceptually related, they represent distinct facets of mental toughness. These findings align with contemporary theoretical frameworks that conceptualize mental toughness as a multifaceted construct comprising distinct but interrelated psychological components (Crust, 2007).

The successful validation of the TMTIS-C has important implications for sports psychology practice in China and potentially other Asian countries. The instrument’s focus on behavioral manifestations of mental toughness (active endeavour, stress resistance, and endurance) aligns well with practical applications in coaching and athlete development. Unlike some Western instruments that emphasize cognitive confidence and control, the TMTIS-C’s emphasis on behavioral persistence and stress management may better capture mental toughness as understood within Chinese sporting contexts. This practical orientation is particularly relevant given research demonstrating that behavioral approaches to mental skills training often produce more tangible improvements in athletic performance compared to purely cognitive interventions (Martin et al., 2001).

The validation of the TMTIS-C also contributes to the growing understanding of how cultural factors influence the expression and measurement of psychological constructs in sport. Research has consistently shown that culturally adapted instruments often demonstrate superior psychometric properties and greater practical utility compared to direct translations of Western-developed measures (Hambleton et al., 2005). The TMTIS-C’s focus on behavioral dimensions aligns particularly well with intervention approaches that emphasize skill development and practical application.

The findings also contribute to the ongoing debate regarding the universality versus cultural specificity of mental toughness constructs. While the three-factor structure remained intact across cultural contexts, the high reliability coefficients and strong factor loadings suggest that the TMTIS-C may be particularly well-suited for Chinese populations. This supports arguments for culturally adapted instruments rather than direct translations of Western scales, particularly in cross-cultural research where construct equivalence cannot be assumed. Contemporary approaches to cross-cultural psychology emphasize the importance of emic (culture-specific) approaches to understanding psychological phenomena, suggesting that culturally adapted instruments may provide more valid assessments than universal measures (Berry, 2002).

From a theoretical perspective, validation supports trait-based conceptualizations of mental toughness while acknowledging its multidimensional nature. The stability of the instrument over time (as evidenced by test–retest reliability) supports viewing mental toughness as a relatively stable individual difference, consistent with dispositional theories of personality. However, the moderate inter-factor correlations suggest that mental toughness dimensions may be differentially trainable, supporting intervention approaches that target specific components rather than treating mental toughness as a unitary construct. This perspective aligns with contemporary research demonstrating that different psychological skills respond differently to various intervention approaches, suggesting the need for multifaceted training programs (Vealey, 2007).

The practical implications of this validation extend to youth development in basketball, where mental toughness has been identified as a critical factor in long-term athletic development. Research has demonstrated that early identification and development of psychological skills can significantly impact athletes’ long-term success and wellbeing (Côté and Hay, 2002). The TMTIS-C’s focus on behavioral dimensions makes it particularly suitable for tracking intervention outcomes and informing individualized training programs.

### Limitations and future research directions

Several limitations of this study should be acknowledged. First, the cross-sectional design precludes conclusions about the TMTIS-C’s sensitivity to change over time, its responsiveness to mental skills interventions, or its predictive relationship with performance outcomes. Future longitudinal studies should address these gaps, given that psychological training effects often emerge over extended timeframes (Hatzigeorgiadis et al., 2011).

Second, the sample was restricted to basketball players from Shandong Province, limiting generalisability to other sports, regions, and populations. The team-based nature of basketball may differentially emphasise certain mental toughness components, and regional variation in athletic culture across China means findings may not transfer uniformly. Further validation across different sports, Chinese provinces, and adult populations is warranted. Additionally, psychological constructs may manifest differently across developmental stages (Steinberg, 2013), necessitating validation in adult samples beyond the current secondary school cohort.

Third, measurement invariance across subgroups such as gender and age cohort was not examined. Multi-group CFA testing configural, metric, and scalar invariance would confirm whether the TMTIS-C operates equivalently across different athlete groups—a prerequisite for valid subgroup comparisons (Vandenberg and Lance, 2000). Future studies should address this gap, as mental toughness expression may vary across gender and developmental stage (Hanrahan and Cerin, 2009).

Fourth, test–retest reliability was assessed on a subsample of 55 participants. Although this met *a priori* power requirements (Arifin, 2022), ICC estimates from smaller samples carry wider confidence intervals. Future work should target subsamples of at least 100 participants for greater precision.

Fifth, validity evidence was limited to structural, convergent, and discriminant validity. Criterion-related validity—linking TMTIS-C scores to objective performance indicators, coach evaluations, or competition outcomes—remains to be established and should be prioritised in future research.

Beyond these limitations, several additional directions merit future investigation. Establishing normative data for the TMTIS-C across different age groups, competitive levels, and sport types would substantially enhance its practical utility for coaches and practitioners. The development of sport-specific adaptations for individual and endurance sports—where the psychological demands differ from team-based contexts—represents a further avenue for extending the instrument’s applicability. Finally, cross-cultural validation studies comparing the TMTIS-C with established Western instruments such as the MTQ-48 and SMTQ would help clarify the extent to which mental toughness constructs are universal or culture-specific, contributing to more comprehensive theoretical models of psychological functioning in sport (Schinke and Hanrahan, 2009).

## Conclusion

This study successfully validated the TMTIS-C as a psychometrically sound instrument for assessing mental toughness in young Chinese basketball players. The confirmatory factor analysis supported the three-factor structure with excellent fit indices, while reliability and validity analyses demonstrated robust psychometric properties across all dimensions. The instrument’s cultural appropriateness and strong psychometric performance make it a valuable tool for researchers and practitioners working with Chinese athletic populations. These findings contribute significantly to the cross-cultural understanding of mental toughness measurement and provide a foundation for future research examining mental toughness development and intervention effectiveness in Asian sporting contexts.

## Data Availability

The raw data supporting the conclusions of this article will be made available by the authors, without undue reservation.
