# Supplementary material for: Validation of the Chinese version of trait mental toughness inventory for sport in young basketball players: a confirmatory factor analysis
Source: Front Psychol. 2026 May 21;17:1772600. doi: 10.3389/fpsyg.2026.1772600 (PMC13233186; doi:10.3389/fpsyg.2026.1772600)
Supplement: Supplementary file 1 [file Supplementary_file_1.docx]

Appendix A

| **心理韧性量表中文简体（C-TMTIS）** | | | | | | |
| --- | --- | --- | --- | --- | --- | --- |
| 此部分主要测量您在运动情景下的心理状态，请详细回想您在运动情景中的实际情况，并仔细回答每一道题目。请您针对下列32道题目选出最符合的答案。 | | | | | | |
| 题目 | | 非常不同意 | 不同意 | 无意见 | 同意 | 非常同意 |
| 1 | 训练时我会尽力达到自己所设定的目标。 |  |  |  |  |  |
| 2 | 比赛时不管输赢 ，我都会奋战到底。 |  |  |  |  |  |
| 3 | 训练时我会不断的想去超越自己的体能。 |  |  |  |  |  |
| 4 | 紧张的时候 ，我会有办法马上放松下來。 |  |  |  |  |  |
| 5 | 就算比赛时的干扰很多 ，我也不会分心。 |  |  |  |  |  |
| 6 | 虽然表现的不理想 ，但我对自己还是很有信心。 |  |  |  |  |  |
| 7 | 有时候身上有一些伤痛，我还是会坚持参加训练。 |  |  |  |  |  |
| 8 | 训练时我会一直想去追求进步。 |  |  |  |  |  |
| 9 | 遇到困难时 ，我会保持冷静。 |  |  |  |  |  |
| 10 | 面对挑战时 ，我会很沉稳的接受它。 |  |  |  |  |  |
| 11 | 训练的时候身体常有一些酸痛 ，我都会忍下来。 |  |  |  |  |  |
| 12 | 为了比別人好 ，我会自觉的练习。 |  |  |  |  |  |
| 13 | 比赛时无论如何 ，我会努力的达成自己的目标。 |  |  |  |  |  |
| 14 | 训练是很严厉的 ，我通常会咬紧牙关坚持。 |  |  |  |  |  |
| 15 | 落后时 ，我还是会稳扎稳打。 |  |  |  |  |  |
| 16 | 就算比赛时感觉压力很大 ，我还是会很专心。 |  |  |  |  |  |
| 17 | 虽然身上有一些伤痛 ，我还是会持续的参与训练。 |  |  |  |  |  |
| 18 | 训练时我会努力的去学习新的东西。 |  |  |  |  |  |
| 19 | 训练虽然很辛苦 ，我还是会完全的投入。 |  |  |  |  |  |
| 20 | 尽管在比赛时落后，我通常还是会表现得很有自信。 |  |  |  |  |  |
| 21 | 有压力时 ，我的抗压能力很好。 |  |  |  |  |  |
| 22 | 比赛的时候身体常有一些酸痛 ，我都会忍下来。 |  |  |  |  |  |
| 23 | 训练时我会尽力达到教练的要求。 |  |  |  |  |  |
| 24 | 比赛时无论如何 ，我会付出全力去争取荣誉。 |  |  |  |  |  |
| 25 | 比赛的时候如果受了一点伤 ，我通常会忍下来。 |  |  |  |  |  |
| 26 | 训练虽然很累 ，我会要求自己做好基本动作。 |  |  |  |  |  |
| 27 | 比赛时我通常都会从开始坚持到最后。 |  |  |  |  |  |
| 28 | 虽然有压力，但我通常还是会把一切都控制的很好。 |  |  |  |  |  |
| 29 | 训练是很枯燥的 ，但我还是会坚持下去。 |  |  |  |  |  |
| 30 | 虽然落后但我还是会积极的进攻。 |  |  |  |  |  |
| 31 | 我不会因为落后而觉得很紧张。 |  |  |  |  |  |
| 32 | 训练时虽然很辛苦 ，但我还是会自我要求。 |  |  |  |  |  |

| **Chinese Version of the Mental Toughness Inventory in Sports (C-TMTIS) –English Version** | | | | | | |
| --- | --- | --- | --- | --- | --- | --- |
| **Instructions:** This section assesses your psychological characteristics in sport contexts. Please reflect on your actual experiences during training and competition and respond to each statement carefully. | | | | | | |
| Item | | Strongly Disagree | Disagree | Neutral | Agree | Strongly Agree |
| 1 | During training, I strive to achieve the goals I have set for myself |  |  |  |  |  |
| 2 | During competition, regardless of the outcome, I persist until the end |  |  |  |  |  |
| 3 | During training, I continuously challenge my physical limits |  |  |  |  |  |
| 4 | When I feel nervous, I can quickly regulate myself and relax |  |  |  |  |  |
| 5 | Even when there are many distractions during competition, I remain focused |  |  |  |  |  |
| 6 | Even when my performance is not ideal, I maintain confidence in myself |  |  |  |  |  |
| 7 | When experiencing minor physical discomfort, I remain engaged in training as appropriate |  |  |  |  |  |
| 8 | During training, I consistently strive for improvement |  |  |  |  |  |
| 9 | When facing difficulties, I remain calm |  |  |  |  |  |
| 10 | When facing challenges, I approach them with composure |  |  |  |  |  |
| 11 | During training, I am able to tolerate physical discomfort when appropriate |  |  |  |  |  |
| 12 | To improve my performance, I engage in self-initiated practice |  |  |  |  |  |
| 13 | During competition, I strive to achieve my goals under all circumstances |  |  |  |  |  |
| 14 | Training can be demanding, but I persist through challenges |  |  |  |  |  |
| 15 | When I am behind, I continue to perform steadily and strategically |  |  |  |  |  |
| 16 | Even under high pressure during competition, I remain focused |  |  |  |  |  |
| 17 | When experiencing minor physical discomfort, I continue training within safe limits |  |  |  |  |  |
| 18 | During training, I actively seek to learn new skills or knowledge |  |  |  |  |  |
| 19 | Although training can be exhausting, I remain fully engaged |  |  |  |  |  |
| 20 | Even when I am behind in competition, I maintain confidence |  |  |  |  |  |
| 21 | I cope effectively with pressure |  |  |  |  |  |
| 22 | During competition, I am able to tolerate physical discomfort when appropriate |  |  |  |  |  |
| 23 | During training, I strive to meet my coach’s expectations |  |  |  |  |  |
| 24 | During competition, I give my best effort to achieve success and honor |  |  |  |  |  |
| 25 | If I experience minor physical discomfort during competition, I manage it and continue appropriately |  |  |  |  |  |
| 26 | Even when training is tiring, I ensure proper execution of fundamental skills |  |  |  |  |  |
| 27 | During competition, I persist from beginning to end |  |  |  |  |  |
| 28 | Even under pressure, I am able to maintain control over my performance |  |  |  |  |  |
| 29 | Training can be monotonous, but I remain committed |  |  |  |  |  |
| 30 | Even when behind, I continue to compete actively |  |  |  |  |  |
| 31 | I do not become anxious when I am behind |  |  |  |  |  |
| 32 | Even when training is demanding, I maintain strong self-discipline |  |  |  |  |  |

Note: The TMTIS-C comprises three subscales. Factor membership for all 32 items is as follows:

Positive Effort (16 items): TMTIS1, TMTIS2, TMTIS3, TMTIS8, TMTIS12, TMTIS13, TMTIS14, TMTIS18, TMTIS19, TMTIS23, TMTIS24, TMTIS26, TMTIS27, TMTIS29, TMTIS30, TMTIS32

Antipressure (11 items): TMTIS4, TMTIS5, TMTIS6, TMTIS9, TMTIS10, TMTIS15, TMTIS16, TMTIS20, TMTIS21, TMTIS28, TMTIS31

Endurance (5 items): TMTIS7, TMTIS11, TMTIS17, TMTIS22, TMTIS25
